# Supplementary figures and images for: Microglia have limited influence on early prion pathogenesis, clearance, or replication
Source: PLoS One. 2022 Oct 27;17(10):e0276850. doi: 10.1371/journal.pone.0276850 (PMC9612458; doi:10.1371/journal.pone.0276850)

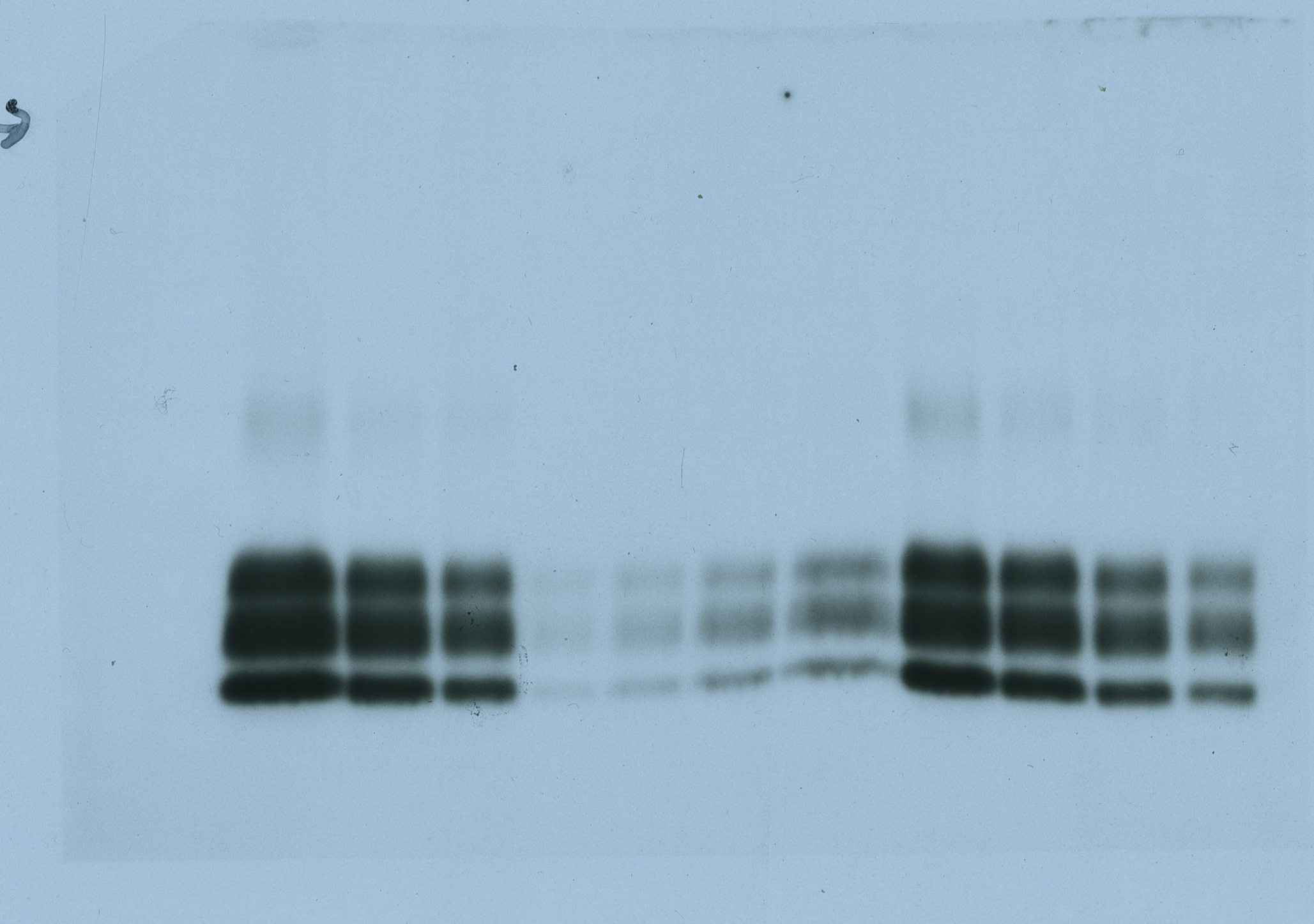

Supplement: S1 Raw image — (TIF) [file pone.0276850.s002.tif]
