# Supplementary material for: Microglia have limited influence on early prion pathogenesis, clearance, or replication
Source: PLoS One. 2022 Oct 27;17(10):e0276850. doi: 10.1371/journal.pone.0276850 (PMC9612458; doi:10.1371/journal.pone.0276850)
Supplement: Attachment — Submitted filename: Response to Reviewers.docx [file pone.0276850.s003.docx]

Reviewer #2:

The paper describes prion replication in the brains of mice whose microglia (MG) has been depopulated (pharmacological treatment using PLX5622 drug) at different stages before or after inoculation with 22L prion strains. The rationale for this approach is that early drug treatment and MG depletion after Prion infection significantly accelerates clinical disease. This results have been obtained earlier in the laboratory and suggest a protective role of MG in the disease course (up to 30 days of life expectancy in this particular study). This result prompted the authors to investigate in further details MG contribution: first their contribution in early disease phase; in a second series of experiments, the authors investigated the effects of repeated MG depopulation during the course of a prion infection. Ultimately, since high PrPC expression levels resulted in accelerated disease but associated less PrPSc depots, the role of basal level of prion expression has been investigated in the context of MG depopulation in either high PrP expressor (tga20 mice) versus wild-type mice.

The main findings of this paper is that MG depletion is of little impact on prion replication, whatever the depopulation occurs in early phase or repeated conditions, or even when high PrPC expression levels are considered.

The experiments are precisely described, and the authors conclude in the absence of MG influence on the course of the prion disease. However, these main results have been obtained in a context where:

- the incubation time is only marginally reduced in treated vs control animals (as compared with the nearly 3 weeks reduction observed in the work by Carroll et al., ref 22);

This is true only for the Tga20 mice that expresses 10 times the PrPC and die from prion disease at 3 times faster than the wildtype mice that were used previously. This is a different model that cannot be directly compared to models previously used.

- only 10-25% of the MG are indeed depopulated as compared with the 90% reduction of the previous study (at least in the last two experiments, cf MS lines 205-6, figure 4 C-E)

The modest reduction to which the review is referring was only true for the attempted MG repopulation studies reported herein. We achieved 64% to 86% reductions (depending on the dpi) in the prion challenge studies associated with Figures 1, 2, and 3, which were comparable reductions to our previous studies using wildtype mice with continual, long-term treatment with PLX5622. In the experimental model outlined in Figure 4, we assessed intermittent pulse PLX5622 treatments using a published method on mice that were in a more advanced disease state. These repopulation studies are not comparable to our previous studies using long-term, continuous PLX5622 treatment.

- the seeding activity, as evaluated using RT-QuIC is statistically the same in both groups.

This set of data suggests that the MG depletion did not occur as it has in the previous report. The only point that is, in my opinion, suggestive of a MG reduction in the treated animals during the first experiment is the staining of TMEM119 (shown as a photo in fig 1), since this labeling is more specific to MG (cf figure 1 panels J and K). However the authors do not present any quantitative data regarding this reduction.

The early PLX5622 treatments that were performed for figures 1, 2, and 3 were as effective as our previously reported studies published in 2018 and 2020, and we assumed our TMEM119 immunohistochemistry would have been convincing enough to demonstrate this point. But the reviewer is correct. We should show this data, and we have incorporated the MG reductions for each of these experimental parameters in figures 1 and 3.

Since MG depletion did not occur as expected, I wonder why the authors did not comment further on the administration process, that may have not been appropriate for the required depletion timing. With respect to the drug administration modes, may the differences between the three experiments account for the differential efficacy of MG depletion? These differences are sufficiently significant to be discussed.

The MG depletion was only ineffective during the studies involving the intermittent PLX5622 treatments associated with Figure 4. We feel we adequately discussed this in the original discussion on lines 311-316 of the original manuscript. The reasons why PLX5622 is less effective at reducing MG in the later stages of disease are unknown, but as we stated in the discussion “Csf1R and Csf1 are increased in the CNS during prion infection and might alter the pharmacological dynamics of PLX5622 treatment.” We refer to this limitation here and in past publications.

I do not completely agree with the initial statement of the discussion in which the authors pledge that “the benefit provided by MG during prion infection likely occurs at the late stages of disease”. This is not demonstrated in this MS, but rather in the previous ref 22.

We respectfully disagree with this statement. Our hypothesis was that MG are able to reduce the amount of infectious prions in the CNS in the early days after inoculation. Using the most sensitive approaches at our disposal, the data support the null hypothesis: that there is no measurable difference in prion amount from day 3 to day 112 post inoculation when MG are reduced. Taking this under consideration we state that these data “support earlier findings” as we interpret the results in the Discussion section.

In the same way, since MG repopulation is proposed to occur after 60 dpi (end of the early PLX5622 treatment), and therefore is suspected to be responsible for the overall lack of efficiency of the treatment, a control experiment in which treatment would have been administered all along should have been set up.

There seems to be some confusion, and the fault is ours. Just to reiterate; the 60-dpi endpoint of treatment that the reviewer is referring (lines 357-358 of the Methods) was used only in the limiting dilution end-point titration experiment as indicated (Figure 1). For the stereotactic experiments (Figure 2 and 3a) PLX5622 was initiated at day -7 and stopped at day +7 as indicated (line 359 of the Methods). For the longer experiments (Figure 3b) PLX5622 was initiated at day -7 continued until mice were euthanized as indicated (lines 359-360 of the Methods). The repopulation experiments in figure 4 PLX5622 was fed in three variations of one-week intervals as indicated on line 361 and the schematic of the experimental design in figure 4a. With so many different treatment protocols to address the various experiments, the confusion is understandable. To help alleviate this, we have added the treatment protocols found in the Methods to the figure legends of figures 1, 2, and 3. We hope this will aid the reader in understanding the various protocol used to assess the influence of MG on prion clearance.

The proper comparative controls for each experiment were performed (in agreement with reviewer 1). Performing continual treatment with PLX5622 on a large cohort of mice, which constitutes our previous studies published 2018 and 2020, would not inform or influence these results. We have shown herein (as have others) that PLX5622 treatment is effective at reducing MG (addressed in the revision). The only issue, which we addressed in the original version, is with short intermittent PLX5622 treatments.

Minor comment :

The meaning of sentence L173-176 is difficult to catch.

We have rewritten this sentence to clarify the statement and can be found on lines 181-185 of the revised manuscript.

In the RT-QuIC M&M section, since it is mentioned for every other component, please precise PrP concentration.

We have added the PrP concentration per the reviewer’s request, which is 0.1mg/ml final concentration.
